# Effect of a Pediatric Early Warning System Educational Program on Nurses’ Capacity to Detect Clinical Deterioration: A Quasi‐Experimental Study

**DOI:** 10.1155/jonm/5109940

**Published:** 2026-10-19

**Authors:** Zeinab Elsayed Hafez Elsayed, Rabia H. Haddad, Ashraf J. Abuejheisheh, Amal A. Elbilgahy, Hend Reda Ali Elkest, Sohaib Abdelhaq Aboskeen, Mai Hassan Hassan El-Sharkawy

**Affiliations:** ^1^ Pediatric Nursing Faculty of Nursing Tanta University Tanta Egypt tanta.edu.eg; ^2^ Pediatric Nursing Department Faculty of Nursing New Mansoura University Mansoura Egypt nmu.edu.eg; ^3^ Department of Nursing Faculty of Nursing Philadelphia University Amman Jordan philau.edu; ^4^ Nursing Department Faculty of Health Professions Al-Quds University Jerusalem State of Palestine alquds.edu; ^5^ Pediatric Nursing Faculty of Nursing Northern Border University Arar Saudi Arabia nbu.edu.sa; ^6^ Pediatric Nursing Department Faculty of Nursing Mansoura University Mansoura Egypt mans.edu.eg; ^7^ Community Health Nursing Tanta University Tanta Egypt tanta.edu.eg; ^8^ Pediatric Nursing Al Rayan National College of Nursing Al Rayan National Colleges Al-Madinah Al-Munawarah Saudi Arabia

**Keywords:** clinical deterioration, nurses capacity, nursing education, pediatric early warning system

## Abstract

**Background:**

Pediatric early warning system (PEWS) supports the early recognition of clinical deterioration in hospitalized children. However, inadequate nurse training may limit their effective implementation in clinical practice. This study evaluated the effect of a PEWS‐based educational program on nurses’ knowledge and clinical performance in a pediatric acute care setting.

**Methods:**

A quasi‐experimental study design was conducted among the pediatric nurses working in Tanta University Hospital and Kafr El‐Sheikh Main University Hospital. A convenience sample was selected from the target population, amounting to 114 pediatric nurses, who were divided into intervention and control groups. The data were collected using the Pediatric Early Warning Signs Questionnaire, Modified Pediatric Early Warning Score, and British Columbia PEWS observational checklist. The outcome variables were measured at baseline, immediately after the intervention, and during the follow‐up period. Descriptive statistics, chi‐square or Fisher’s exact test, independent samples *t*‐test, Pearson’s correlation, and multiple linear regression were performed to analyze the data using SPSS statistical software. The statistical significance level was set at *p* < 0.05.

**Results:**

Baseline evaluation showed no statistically significant variations between intervention and control groups among theoretical knowledge and clinical performance. Immediately postintervention, high knowledge levels were attained by 94.7% of nurses in the intervention group versus 24.6% in the control group (*p* < 0.001), while satisfactory clinical performance was observed in 91.2% and 22.8% of nurses, respectively (*p* < 0.001). At follow‐up, high knowledge levels were observed in 87.7% of nurses in the intervention group compared with 10.5% in the control group (*p* < 0.001), and satisfactory clinical performance was observed in 87.7% and 19.3%, respectively (*p* < 0.001). In the intervention group, the level of education and years of experience were significantly associated with both knowledge and clinical performance levels at follow‐up. Knowledge and clinical performance scores were positively correlated in the intervention group (*r* = 0.317, *p* = 0.016).

**Conclusion:**

The findings suggest that structured PEWS education can improve knowledge of clinical deterioration and clinical performance in applying PEWS assessments among pediatric nurses, with improvements largely maintained at follow‐up. Incorporating structured PEWS education into continuing nursing education may strengthen nurses’ capacity to respond to clinical deterioration in pediatric acute care settings.

**Implications for Nursing Management:**

Nursing managers should consider integrating structured PEWS education into workforce‐development programs to strengthen nurses’ knowledge and clinical performance in recognizing pediatric deterioration and support timely escalation of care.

**Highlights**
• PEWS‐based education significantly improved pediatric nurses’ knowledge of clinical deterioration recognition.
• Nurses receiving the intervention demonstrated substantially higher clinical performance than controls.
• Educational level and years of experience were significant predictors of knowledge and practice outcomes.
• Structured PEWS training can strengthen nursing workforce capacity and support patient safety in pediatric acute care settings.

## 1. Background

Egypt is a lower‐middle‐income country, according to the World Bank classification, where children constitute approximately one‐third of the population. According to the United Nations Children’s Fund [1], approximately 33.5% of Egypt’s population is younger than 18 years of age. According to the reported estimates, although child health indicators have improved substantially over recent decades, under‐five mortality in Egypt remains an important public health concern, with the mortality rate for children under five measured at approximately 17 deaths per 1000 live births in 2023 [1, 2]. To maintain these developments, it is necessary to implement specific actions continually to balance the existing limitations in the context of limited resources. Therefore, analyzing the characteristics and outcomes of children who were admitted to the pediatric intensive care unit (PICU) may contribute to the improvement of future healthcare policies [3]. Pediatric acute care units are specialized hospital departments that care for sick or injured children who do not require admission to the PICU. They provide healthcare supervision and nursing services to youths who suffer from severe infections, asthma, postsurgery conditions, or have been injured in an accident. This facility aims to help such patients receive comprehensive treatment and continuous observation around the clock. It also helps ensure that the children are provided with acute care and timely intervention if their conditions worsen. In general, pediatric acute care units offer care and treatment for sick infants and children, thus bridging the gap between general and intensive care for the youths [4].

The nurses play a very important role because they provide care for acute care child patients who mostly require emergency care to deal with fatal and lethal conditions. They offer medication, insert IV lines, take vital signs, and perform comprehensive assessments to make sure that appropriate and expedited measures are taken to stabilize the patient or reduce the severity and progression of the illness [5]. Pediatric early warning system (PEWS) is a nurse‐based algorithm that uses vital signs and clinical findings to identify patients who are at risk for physiological decline. This decline is defined as a downward change in a patient’s condition that increases the risk of organ failure, prolonged hospitalization, adverse disease progression, or death. The use of PEWS helps in early recognition of such cases by clinicians, thus reducing emergency pediatric intensive care admission, in‐hospital cardiac arrests, and mortality in these settings [6].

The scale typically assesses a variety of factors such as heart rate, respiratory rate, blood pressure, level of consciousness, respiratory effort, capillary refill time, and the need for oxygen. Each of these variables is adjusted for the patient’s age and scored according to their distance from the normal value. If the total score of these metrics reaches a predetermined threshold, or demonstrates an increasing trend, this triggers an immediate reassessment or rapid response team activation. The implementation of this approach eliminates ambiguity in communication between the members of the multiprofessional team caring for the child. It provides nurses with objective justification for escalation and facilitates consistent reporting while reducing the risk of delayed care [7].

Even if the effectiveness of PEWS has been proven, the success of the implementation of these systems is primarily determined by nurses’ knowledge, skills, and self‐confidence in providing care. Indeed, previous studies have shown that training courses on PEWS helped nurses raise awareness and properly respond to the signs of clinical deterioration of pediatric patients. For instance, Abdala et al. [8] found that the use of evidence‐based education improved the nurses’ ability to identify pediatric deterioration, and confirmed that education on PEWS enhanced nurses’ knowledge and improved clinical outcomes in pediatric patients with heart diseases who were operated on [9, 10]. Similarly, Ebraheim et al. [11] and Al Zaher et al. [12] highlighted the importance of ongoing education and competency development in sustaining PEWS implementation and improving pediatric patient outcomes. Collectively, these findings indicate that educational preparation is a fundamental component of successful PEWS implementation and provide a strong rationale for evaluating the effectiveness of a PEWS‐based educational program in pediatric acute care settings.

### 1.1. Significance of the Study

Globally, over six million children die every year from preventable causes. Of these, critical conditions that are both reversible and life‐threatening, such as sepsis and acute respiratory failure, contribute to nearly a third of under‐five mortalities, imposing an enormous burden on low‐ and lower‐middle‐income countries. In hospitals, poor initial assessment, inadequate monitoring, and delayed response to clinical deterioration contribute to poor patient outcomes and mostly arise due to the lack of standardized procedures for early recognition of such deterioration [13]. In the pediatric acute care setting, the PEWS scale is a critical clinical tool utilized to detect indicators of deterioration in a child’s condition [14]. This innovative approach to nursing enables staff to identify small physiological changes in patients’ state that signal the possibility of imminent clinical deterioration, thus making it possible to intervene promptly and prevent an emergency if it arises [15]. Development of nurses’ skills in this regard will allow for a decrease in morbidity and mortality rates in pediatric populations, a decrease in length of hospital stays, and achieve better results of treatment [12]. Thus, the objective of this study is to assess how effective the use of PEWS‐based training is in relation to nurses’ performance in clinical practice.

Despite some evidence of the benefits of using PEWS, significant knowledge gaps exist in relation to the extent to which structured educational interventions can help develop and maintain knowledge and clinical performance of nurses in relation to the proper application of PEWS in acute care settings [11, 12]. Most of the available literature mainly focuses on the implementation or validation of PEWS, whereas not many studies have been dedicated to assessing the educational efficiency of training aimed at helping nurses become competent in using PEWS because of their long‐term retention of PEWS.

### 1.2. Aim of the Study

The aim of the study was to evaluate the effect of a structured PEWS educational program on nurses’ knowledge and clinical performance in acute care settings.

## 2. Materials and Methods

### 2.1. Study Design

A quasi‐experimental research design was utilized in this study. PEWS was introduced as part of the educational intervention and was not routinely implemented as a mandatory institutional practice for the participating nurses before completion of the intervention.

### 2.2. Participants and Setting

The study was carried out at the Pediatric Medical Department Units and Acute Care Settings at the Tanta Main University Hospital in Tanta City and the Kafr El‐Sheikh Main University Hospital in Kafr El‐Sheikh City, Egypt. Both hospitals are affiliated with the Ministry of Higher Education and Scientific Research.

A total of 114 pediatric nurses who were working in acute care settings in Tanta Main University Hospital and Kafr El‐Sheikh Main University Hospital were recruited through convenience sampling. Data collection extended from July 2025 to January 2026. Out of this amount, 74 pediatric nurses were employed in the Tanta University Main Hospital, while the Kafr El‐Sheikh Main University Hospital during the study included 40 pediatric nurses. In each hospital studied, the participants were divided into two groups: study group and control group. In the Tanta University Main Hospital, the study group comprised 37 nurses who were trained in the PEWS method and 37 nurses who did not undergo such training. In the Kafr El‐Sheikh Main University Hospital, 20 pediatric nurses took part in the study group and the control group, respectively. All the participants provided nursing services that directly relate to the identification and management of deterioration in pediatric patients. Therefore, only nurses working in these clinical settings were considered eligible for participation. A convenience sampling approach was used to recruit eligible nurses who were available during the study period. For descriptive and categorical analyses, participants’ ages were grouped into < 25, 25–30, and > 30 years to represent relatively younger, intermediate, and older participants within the age distribution of the study sample. These categories were study‐specific analytical groupings and were not based on established clinical age thresholds; therefore, findings based on these categories were interpreted cautiously. Age was also analyzed descriptively as a continuous variable using the mean and standard deviation.

#### 2.2.1. Inclusion and Exclusion Criteria

Pediatric nurses will be eligible to participate in the study if they meet the following criteria:

1. Working as a staff nurse providing direct bedside care in any of the pediatric acute, surgical, or medical wards at the selected healthcare facilities.
2. Employed in the current pediatric unit for at least 6 months prior to the study’s start.
3. Willing to participate in the study and sign the informed consent form.
4. Available to attend the educational sessions and complete the assessments during the data collection period.

However, nurses who meet any of the following criteria will be excluded from the study:

1. If employed in a nonclinical capacity, for instance, a supervisory or teaching appointment, and have no direct patient care duties.
2. On maternity leave, annual leave, sick leave, or any other prolonged authorized absences during the study period.
3. Completed a formal PEWS training course in the past 12 months.
4. Declined to participate in the study or withdrew their consent before completing the postintervention assessments.

### 2.3. Measures

#### 2.3.1. Tool I: PEWS Questionnaire

The PEWS Questionnaire was developed by researchers following a comprehensive review of recent literature [16–18]. In Arabic, the tool has been designed with simplicity so that the nursing personnel taking part in the study can understand the contents of the instrument easily. The instrument consists of two sections, the first of which contains questions related to the demographic details of the participants such as their age, gender, education, work experience, job position, and previous training in capturing pediatric early warning signs. The second part tests the respondents’ knowledge about PEWS as well as clinical deterioration. This section investigates concepts such as the definition of PEWS as well as its aims and significance, goals, and components of the system, and the definition of clinical deterioration, its causes, and symptoms. Three types of scores were given based on the replies, meaning that two points were allotted for an accurate and comprehensive answer, one point for a partially correct answer, while no points were given for a wrong answer or in case of an uncertain reply. The total score for knowledge was categorized into three groups, which were: less than 80%—low level of knowledge, 80%–90%—moderate level of knowledge, and 90%–100%—high level of knowledge.

#### 2.3.2. Tool (II): The Modified Pediatric Early Warning Score

The pediatric early warning score originally conceptualized by Monaghan [19] and subsequently adapted by Romero et al. [20] was employed to support the early identification of clinical decline in pediatric patients. The instrument evaluates three physiological domains across 10 items: behavior (2 items), cardiovascular status (3 items), and respiratory function (5 items). Individual parameters are rated on a four‐point scale from 0 (*none*) to 3 (*severe*), yielding a cumulative domain‐based score between 0 and 9. Furthermore, an additional 2 points are added to the total if the child receives nebulizer therapy every 15 min or presents with persistent postoperative emesis. Each subscale follows a uniform scoring rubric: none (0), mild (1), moderate (2), and severe (3). The final composite score determines the overall clinical risk classification as follows: non/low risk (0–2), mild (3), moderate (4), and sever/high risk (> 5). See Table 1.

**TABLE 1:** The modified pediatric early warning score.

Components	0	1	2	3
Behavior	Playing/appropriate; eating	Sleeping	Irritable	Lethargic, confused, or reduced response to pain
Cardiovascular	Pink; capillary refill 1–2 s	Pale; capillary refill 3 s	Gray; capillary refill 4 s OR tachycardia 20 beats/min above the normal rate	Gray/mottled; capillary refill ≥ 5 s OR tachycardia 30 beats/min above the normal rate OR bradycardia
Respiratory	Within normal parameters; no retractions	> 10 above normal parameters; use of accessory muscles	> 20 above normal parameters; retractions	5 below normal parameters with retractions and grunting

*Note:* Scores for each domain range from 0 (*none*) to 3 (*severe*). The total PEWS score ranges from 0 to 9. An additional 2 points are added if the child receives nebulizer therapy every 15 min or has persistent postoperative emesis. Higher scores indicate greater clinical deterioration risk.

Abbreviation: PEWS, Pediatric Early Warning System.

#### 2.3.3. Tool III: British Columbia Pediatric Early Warning System (BC PEWS) Observational Checklist

The observational checklist used in this study was developed based on BC PEWS, developed by Child Health BC to support the early recognition and management of clinical deterioration in pediatric patients [21]. This tool was created to assess the appropriateness of pediatric vital signs assessment that aids in identifying early signs of physical deterioration in children and thus allows for timely intervention. The checklist includes such parameters as respiratory rate, oxygen saturation (now obtained through pulse oximetry), blood pressure, capillary refill, skin color, temperature, neuro‐vital signs, spinal cord, and neurovascular assessment.

Each step of nursing practice was scored dichotomously: “done” was assigned a score of 1, while “not done” was scored as 0. The total practice score was then classified as follows: unsatisfactory: < 80%, satisfactory: 80%–100%. This structured approach ensures systematic evaluation of nursing performance in pediatric acute care settings and supports adherence to evidence‐based standards for early detection of clinical deterioration.

#### 2.3.4. Content Validity and Reliability

To evaluate content validity, relevance, and item clarity, the research instruments were reviewed by a panel of five pediatric nursing experts. The calculated content validity index (CVI) across all scale items reached 94%. Reliability analysis demonstrated strong internal consistency for the instruments, yielding a Cronbach’s alpha coefficient of 0.891.

#### 2.3.5. Pilot Study

To evaluate the operational feasibility, clarity, and practical application of the research instruments, a pilot trial was executed on 10% of the calculated sample size. Refinements were integrated based on the preliminary feedback, and the participating cohort was subsequently omitted from the main study analysis.

### 2.4. Data Collection

The data were collected by using three study tools. The participating nurses completed Tool I (PEWS Questionnaire) in the clinical setting in the presence of the researchers to ensure its completeness. Tool II (modified pediatric early warning score) was used to assess patients’ vital signs and behavioral observations for early detection of clinical deterioration. The researchers used Tool III (BC PEWS observational checklist) to assess nurses’ clinical practice regarding PEWS.

At Tanta and Kafr El‐Sheikh Main University Hospitals, nurses received a PEWS‐based educational program in the intervention group. The nurses were divided into eight subgroups. The program consisted of four sessions, delivered over the course of two weeks (two sessions/week), 30–45 min each session, which included lectures, video demonstration, and discussion. Content included PEWS principles, recognition of clinical deterioration, physiological parameters, scoring and interpretation, documentation, and appropriate nursing interventions.

Data were collected at baseline, immediately postintervention, and 1 month later by the same study tools. The nurses in the control group continued their routine practice and did not receive any training related to PEWS during the study period. They performed the same assessments at three points. The educational materials of PEWS were provided to the control group after finishing the study.

### 2.5. Ethical Considerations

The Faculty of Nursing at Tanta University granted ethical approval to conduct the research, and the relevant stakeholders from the pediatric medical departments of Tanta University Hospital and Kafr El‐Sheikh Main University Hospital approved the study after the research scope and objectives were explained. Ethical approval for the research was acquired from the Scientific Research Ethics Committee at the Faculty of Nursing in Tanta University (Approval number: 736‐7‐2025). There were no physical risks, discomforts, or strains for participants in the course of the research. The ethical principles of privacy and confidentiality of participants and their information were met. Prior to the data collection process, written informed consent was obtained from each nurse participating in the research, along with information about the voluntary nature of their participation in the process and their right to withdraw from it at any point in time without any consequences.

### 2.6. Statistical Analysis

Statistical analysis was conducted using SPSS Statistics software (Version 26). Frequency distribution and percentage of categorical variables were calculated, while continuous data were presented in terms of mean along with standard deviation (mean ± SD). Prior to carrying out parametric analysis, the distribution of continuous data was tested for normality. Group comparisons for categorical parameters were conducted using the chi‐square test or Fisher’s exact test, depending on expected cell counts, whereas continuous measures were compared using the independent samples *t*‐test.

Participants’ knowledge and clinical performance metrics were evaluated across three timeline intervals: baseline, immediately postintervention, and follow‐up. Cross‐sectional differences in categorical proficiency levels between the two groups were evaluated using chi‐square or Fisher’s exact tests at each timeframe, whereas mean differences in overall knowledge and practice scores were tested via independent samples *t*‐tests. Longitudinal trends in continuous score outcomes across the three assessment points were analyzed using two‐way repeated‐measures analysis of variance (ANOVA), specifying time as the within‐subject factor and group assignment as the between‐subject factor to evaluate the main effects of time and group, as well as the group × time interaction effect.

Bivariate associations between nurses’ baseline demographic profiles and categorical competency classifications at follow‐up were determined using chi‐square or Fisher’s exact tests. Pearson’s correlation coefficient (*r*) was applied to assess the relationship between follow‐up PEWS knowledge and clinical performance scores. Subsequently, multiple linear regression models were executed within the intervention cohort to determine independent predictors of follow‐up PEWS knowledge and performance levels. Model estimates were reported as unstandardized coefficients (B), standard errors (SE), standardized coefficients (*β*), 95% confidence intervals (CI), and corresponding *p* values. All hypotheses were evaluated using two‐tailed tests, setting the threshold for statistical significance at *p* < 0.05.

## 3. Results

The study included 114 nurses, with 57 participants in each group. Baseline demographic characteristics were comparable between groups (Table 2). Mean age was 28.10 ± 2.20 years in the control group and 28.50 ± 2.50 years in the intervention group (*p* = 0.288).

**TABLE 2:** Baseline characteristics of participants by study group.

Characteristic	Control (*n* = 57)		Intervention (*n* = 57)		*χ* ^2^/*t*	*p*
	*n*	%	*n*	%		
Age (years)					4.56	0.103
< 25	7	12.30	5	8.80		
25–30	40	70.20	32	56.10		
> 30	10	17.50	20	35.10		
Age (continuous)	28.10 ± 2.20		28.50 ± 2.50		1.07	0.288
Gender					0.04	0.845
Male	20	35.10	21	36.80		
Female	37	64.90	36	63.20		
Educational level					0.80	0.372
Diploma in nursing	11	19.30	15	26.30		
Bachelor of nursing	46	80.70	42	73.70		
Experience (years)					1.41	0.494
< 5	13	22.80	18	31.60		
5–10	36	63.20	30	52.60		
> 10	8	14.00	9	15.80		
Experience (continuous)	5.80 ± 2.80		5.90 ± 2.80		0.10	0.921
Current occupation					0.80	0.372
Nursing specialist	46	80.70	42	73.70		
Nursing technician	11	19.30	15	26.30		

*Note:* Categorical variables are presented as *n* (%). Continuous variables are presented as mean ± SD. *t*, independent‐samples *t*‐test; χ^2^, chi‐square test.

Table 3 and Figure 1 present nurses’ knowledge levels regarding pediatric early warning signs and clinical deterioration across the three assessment points. At baseline, the distribution of knowledge levels did not differ significantly between the intervention and control groups (χ^2^ = 0.38, *p* = 0.829). Postintervention, 94.70% of nurses in the intervention group had a high knowledge level compared with 24.60% in the control group (χ^2^ = 59.33, *p* < 0.001). At follow‐up, 87.7% of nurses in the intervention group had a high knowledge level compared with 10.50% in the control group (χ^2^ = 68.30, *p* < 0.001). Mean total knowledge scores were also higher in the intervention group than in the control group at postintervention (24.50 ± 1.00 vs. 19.70 ± 4.30, *p* < 0.001) and follow‐up (23.90 ± 2.50 vs. 19.00 ± 3.90, *p* < 0.001), whereas no significant between‐group difference was observed at baseline (16.70 ± 3.60 vs. 17.40 ± 3.40, *p* = 0.331).

**TABLE 3:** PEWS knowledge levels across study assessments.

Assessment	Outcome	Control (*n* = 57)		Intervention (*n* = 57)		*χ* ^2^/*t*	*p*
		*n*	%	*n*	%		
Preintervention	Low	47	82.50	49	86.00		
	Moderate	7	12.30	5	8.80		
	High	3	5.30	3	5.30	0.38	0.829

Postintervention	Low	26	45.60	0	0.00		
	Moderate	17	29.80	3	5.30		
	High	14	24.60	54	94.70	59.33	< 0.001

Follow‐up	Low	31	54.40	3	5.30		
	Moderate	20	35.10	4	7.00		
	High	6	10.50	50	87.70	68.30	< 0.001

Preintervention	Total score	17.40 ± 3.40		16.70 ± 3.60		0.98	0.331

Postintervention	Total score	19.70 ± 4.30		24.50 ± 1.00		8.12	< 0.001

Follow‐up	Total score	19.00 ± 3.90		23.90 ± 2.50		7.89	< 0.001

*Note:* Categorical variables are presented as *n* (%) and continuous variables as mean ± SD. *t*, independent samples *t*‐test; *χ* ^2^, chi‐square test.

**FIGURE 1:**
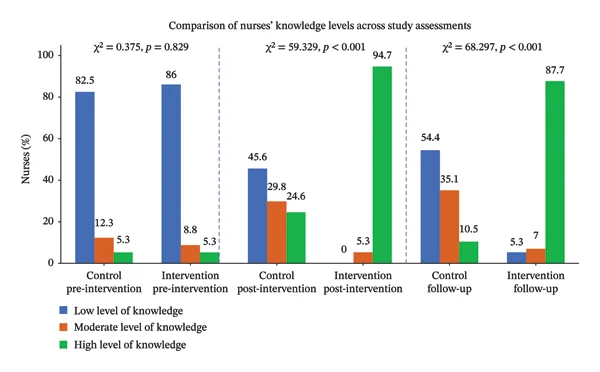
Nurses’ knowledge levels regarding pediatric early warning signs and clinical deterioration.

Table 4 and Figure 2 present nurses’ clinical performance in applying the BC PEWS observational checklist across the three assessment points. At baseline, the distribution of clinical performance levels did not differ significantly between the intervention and control groups (χ^2^ = 2.17, *p* = 0.141), with satisfactory performance observed in 7.00% and 15.80% of nurses, respectively. Postintervention, satisfactory performance was observed in 91.20% of nurses in the intervention group compared with 22.8% in the control group (χ^2^ = 54.44, *p* < 0.001). At follow‐up, the corresponding proportions were 87.70% and 19.30%, respectively (χ^2^ = 53.63, *p* < 0.001). Mean clinical performance scores were higher in the intervention group than in the control group at postintervention (33.30 ± 2.70 vs. 25.00 ± 6.20, *p* < 0.001) and follow‐up (32.30 ± 3.50 vs. 25.80 ± 6.10, *p* < 0.001), whereas no significant between‐group difference was observed at baseline (23.40 ± 5.30 vs. 24.20 ± 5.80, *p* = 0.476).

**TABLE 4:** Clinical performance levels and total performance scores across study assessments.

Assessment	Outcome	Control (*n* = 57)		Intervention (*n* = 57)		*χ* ^2^/*t*	*p*
		*n*	%	*n*	%		
Preintervention	Unsatisfactory	48	84.20	53	93.00		
	Satisfactory	9	15.80	4	7.00	2.17	0.141

Postintervention	Unsatisfactory	44	77.20	5	8.80		
	Satisfactory	13	22.80	52	91.20	54.44	< 0.001

Follow‐up	Unsatisfactory	46	80.70	7	12.30		
	Satisfactory	11	19.30	50	87.70	53.63	< 0.001

Preintervention	Total score	24.20 ± 5.80		23.40 ± 5.30		0.71	0.476

Postintervention	Total score	25.00 ± 6.20		33.30 ± 2.70		9.22	< 0.001

Follow‐up	Total score	25.80 ± 6.10		32.30 ± 3.50		6.88	< 0.001

*Note:* Values are presented as *n* (%) or mean ± SD, as appropriate; χ^2^ = chi‐square test; *t* = independent‐samples *t*‐test. *p* < 0.05 was considered statistically significant.

Abbreviation: SD, standard deviation.

**FIGURE 2:**
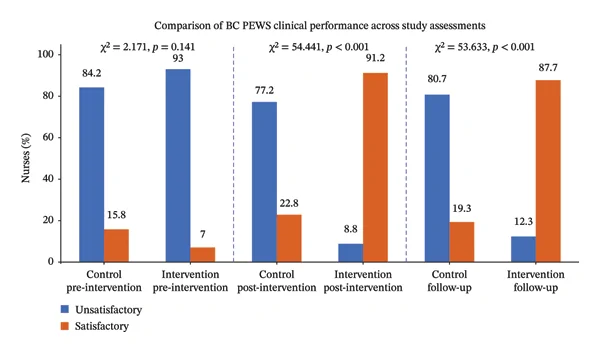
Nurses’ clinical performance levels based on the British Columbia pediatric early warning system observational checklist.

Table 5 presents the associations between nurses’ baseline sociodemographic profiles and their PEWS knowledge levels at the follow‐up assessment. Within the control cohort, age demonstrated a statistically significant association with knowledge outcomes (χ^2^ = 12.52, *p* = 0.014), whereas gender, educational attainment, professional experience, and job role showed no significant relationships. Conversely, within the intervention group, follow‐up knowledge levels were significantly associated with both educational level (χ^2^ = 10.59, *p* = 0.005) and experience years (χ^2^ = 17.29, *p* = 0.002). However, no significant associations were detected regarding age, gender, or current position in this cohort.

**TABLE 5:** Associations between nurses’ characteristics and PEWS knowledge levels at follow‐up.

Assessment	Control (*n* = 57)								Intervention (*n* = 57)							
	Low		Moderate		High		*χ* ^2^/Fisher	*p*	Low		Moderate		High		*χ* ^2^/Fisher	*p*
	*n*	%	*n*	%	*n*	%			*n*	%	*n*	%	*n*	%		
Age (years)							12.52	0.014							2.28	0.685
< 25	2	6.50	3	15.00	2	33.30			0	0.00	0	0.00	5	10.00		
25–30	19	61.30	17	85.00	4	66.70			1	33.30	2	50.00	29	58.00		
> 30	10	32.30	0	0.00	0	0.00			2	66.70	2	50.00	16	32.00		
Gender							0.41	0.813							4.66	0.098
Male	12	38.70	6	30.00	2	33.30			0	0.00	0	0.00	21	42.00		
Female	19	61.30	14	70.00	4	66.70			3	100.00	4	100.00	29	85.00		
Educational level							1.85	0.396							10.59	0.005
Diploma in nursing	6	19.40	5	25.00	0	0.00			3	100.00	2	50.00	10	20.00		
Bachelor of nursing	25	80.60	15	75.00	6	100.00			0	0.00	2	50.00	40	80.00		
Experience (years)							5.51	0.238							17.29	0.002
< 5	10	32.30	2	10.00	1	16.70			3	100.00	4	100.00	11	22.00		
5–10	18	58.10	15	75.00	3	50.00			0	0.00	0	0.00	30	60.00		
> 10	3	9.60	3	15.00	2	33.30			0	0.00	0	0.00	9	18.00		
Current occupation							1.85	0.396							2.85	0.241
Nursing specialist	25	80.60	15	75.00	6	100.00			3	100.00	4	100.00	35	70.00		
Nursing technician	6	19.40	5	25.00	0	0.00			0	0.00	0	0.00	15	30.00		

*Note:* Categorical variables are presented as *n* (%) and continuous variables as mean ± SD; *χ* ^2^, chi‐square test. Fisher’s exact test was used where appropriate. *p* < 0.05 was considered statistically significant.

Table 6 presents the associations between nurses’ demographic characteristics and clinical performance levels at follow‐up. In the control group, no statistically significant associations were observed between clinical performance level and age, gender, educational level, years of experience, or current occupation. In the intervention group, clinical performance level was significantly associated with educational level (χ^2^ = 14.52, *p* < 0.001) and years of experience (χ^2^ = 10.89, *p* = 0.004), whereas no significant associations were observed with age, gender, or current occupation.

**TABLE 6:** Associations between nurses’ characteristics and clinical performance levels at follow‐up.

Assessment	Control (*n* = 57)						Intervention (*n* = 57)					
	Unsatisfactory		Satisfactory		*χ* ^2^/Fisher	*p*	Unsatisfactory		Satisfactory		*χ* ^2^/Fisher	*p*
	*n*	%	*n*	%			*n*	%	*n*	%		
Age (years)					3.34	0.188					1.13	0.569
< 25	6	13.00	1	9.10			0	0.00	5	10.00		
25–30	30	65.20	10	90.90			5	71.40	27	54.00		
> 30	10	21.70	0	0.00			2	28.60	18	36.00		
Gender					0.01	0.921					1.75	0.187
Male	16	37.80	4	36.40			1	14.30	20	40.00		
Female	30	65.20	7	63.60			6	85.70	30	60.00		
Educational level					0.56	0.455					14.52	< 0.001
Diploma in nursing	8	17.40	3	27.30			6	85.70	9	18.00		
Bachelor of nursing	38	82.60	8	72.70			1	14.30	41	82.00		
Experience (years)					4.03	0.134					10.89	0.004
< 5	13	28.30	0	0.00			6	85.70	12	24.00		
5–10	27	58.70	9	81.80			1	14.30	29	58.00		
> 10	6	13.00	2	18.20			0	0.00	9	18.00		
Current occupation					3.26	0.071					0.60	0.440
Nursing specialist	35	76.10	11	100.00			6	85.70	36	72.00		
Nursing technician	11	23.90	0	0.00			1	14.30	14	25.00		

*Note:* Values are presented as *n* (%). χ^2^ = chi‐square test. Fisher’s exact test was used where appropriate. *p* < 0.05 was considered statistically significant.

Table 7 presents the correlation between nurses’ PEWS knowledge and clinical performance scores at follow‐up. In the intervention group, a statistically significant positive correlation was observed between knowledge and clinical performance scores (*r* = 0.32, *p* = 0.016). In the control group, the correlation between knowledge and clinical performance scores was not statistically significant (*r* = 0.21, *p* = 0.123).

**TABLE 7:** Correlation between PEWS knowledge and clinical performance at follow‐up.

Group	*r*	*p*
Control (*n* = 57)	0.21	0.123
Intervention (*n* = 57)	0.32	0.016

*Note: r* = correlation coefficient. *p* < 0.05 was considered statistically significant.

Table 8 presents the multiple linear regression analysis of factors associated with nurses’ PEWS knowledge scores at follow‐up in the intervention group. Educational level (*β* = 0.26, *p* = 0.032), years of experience (*β* = 0.22, *p* < 0.001), and BC PEWS clinical performance (*β* = 0.02, *p* = 0.027) were significantly associated with knowledge scores. Age, gender, and current occupation were not statistically significantly associated with knowledge scores.

**TABLE 8:** Predictors of PEWS knowledge score at follow‐up.

Predictor	*B*	SE	β	*t*	*p*	95% CI
Constant	25.14	2.98		8.45	< 0.001	[19.17, 31.11]
Age	−0.27	0.55	−0.06	−0.48	0.631	[−1.38, 0.84]
Gender	−0.82	0.70	−0.16	−1.18	0.245	[−2.55, 0.35]
Educational level	1.19	0.10	0.26	1.99	0.032	[−2.25, 1.92]
Experience (years)	1.65	0.11	0.22	2.58	< 0.001	[−1.06, 1.02]
BC PEWS observational checklist score	1.17	0.04	0.02	2.16	0.027	[−2.25, 1.92]
Current occupation	0.02	0.52	−0.01	−2.04	0.972	[−0.33, 2.82]

*Note:* B = unstandardized regression coefficient; β = standardized regression coefficient. *p* < 0.05 was considered statistically significant.

Abbreviations: CI, confidence interval; SE, standard error.

Table 9 presents the multiple linear regression analysis of factors associated with nurses’ clinical performance scores on the BC PEWS observational checklist at follow‐up. PEWS knowledge (*β* = 0.28, *p* = 0.032), educational level (*β* = 0.17, *p* = 0.028), and years of experience (*β* = 0.15, *p* = 0.031) were significantly associated with clinical performance scores. Age, gender, and current occupation were not statistically significantly associated with clinical performance scores.

**TABLE 9:** Predictors of clinical performance score at follow‐up.

Predictor	B	SE	Β	*t*	*p*	95% CI
Constant	24.91	5.67		4.40	< 0.001	[13.54, 36.29]
Age	0.02	0.76	0.00	0.03	0.977	[−1.50, 1.55]
Gender	−0.95	1.00	−0.13	−0.95	0.348	[−2.96, 1.06]
Educational level	1.64	0.51	0.17	1.49	0.028	[−2.53, 1.90]
Experience (years)	1.38	0.33	0.15	1.04	0.031	[−1.76, 1.07]
Current occupation	0.32	1.10	0.04	0.29	0.774	[−0.00, 0.77]
PEWS knowledge score	1.38	0.19	0.28	1.99	0.032	[−1.90, 2.53]

*Note:* B = unstandardized regression coefficient; β = standardized regression coefficient. *p* < 0.05 was considered statistically significant.

Abbreviations: CI, confidence interval; SE, standard error.

## 4. Discussion

Pediatric acute care settings require rapid and accurate clinical judgments, particularly in identifying indicators of deterioration among hospitalized children. Nurses play an essential role in this process, as they are often the first to detect subtle physiological and behavioral changes [22, 23]. The PEWS offers an evidence‐based and systematic strategy to enable prompt identification and escalation of care. However, the effectiveness of this system relies on the understanding, confidence, and ability of nurses to use it in their clinical practice [5, 24].

The information provided in Table 2 and Figure 1 indicates that the knowledge of nurses from the intervention group improved as a result of implementing the PEWS training, which also succeeded in maintaining this change over time. The absence of baseline differences between groups suggests that the observed changes can be linked to the intervention itself. Lower baseline knowledge and performance scores in the control group are expected because the subjects had yet to receive any structured PEWS training [8, 9]. The results are consistent with earlier research, which showed that many nurses have insufficient knowledge regarding pediatric deterioration because of a lack of proper formal training and inconsistent levels of experience [9]. Taken together, these results depict that structured interventions help bridge the gap and improve cognitive preparedness.

Consistent with current findings, research on PEWS‐based education yielded similar results regarding nurses’ knowledge and skills with PEWS. The findings of Sridhar et al. [9] highlighted that education in this area led to an increase in the ability of nurses to detect early warning signs of conditions in children following heart surgery. Abdala et al. [8] also showed that scientific education can help develop the ability of nurses to identify issues in children suffering from clinical deterioration. In general, these studies demonstrated that educational interventions can be used to address the existing knowledge gaps relating to pediatric care.

Moreover, Olowo et al. [25] discovered significant initial deficiencies in nurses’ understanding of modified early warning score (MEWS). The results showed that more than 67% of the nurses belonged to the category of novices, as the nurses did not have any formal training (74% of respondents reported no prior training). The study also demonstrated that the knowledge of nurses significantly increased after the training intervention was implemented (Wilcoxon signed‐rank test: *Z* = 7.631, *p* < 0.0001). The enhancement can be explained by utilizing Knowles’s adult learning theory, which stresses the significance of the relevance of the learning process for the learners, independence, and hands‐on experience in ensuring the success of education [26]. These conclusions are supported by numerous studies conducted in Africa and worldwide. For example, the research conducted by Ebraheim et al. [11] revealed that nurses’ skills of recognizing and responding to urgent situations improved considerably as a result of PEWS training in Egypt. What is more, research undertaken by Damayanti et al. [27], Mussa et al. [28], and Saab et al. [29] has proved that training helps to enhance the knowledge and skills of nurses in the area of application of the early warning scoring systems. Overall, all mentioned studies confirm the effectiveness of educational approaches, similar to the approaches used in this observation.

Importantly, follow‐up data demonstrating lasting benefit from the education reinforces the long‐term effect of the intervention. The percentage of nurses in the intervention arm demonstrating high knowledge retention was significantly larger than in the control arm (87.7% vs. 10.5%) (*p* < 0.001), which indicates that the training has helped the clinicians not only learn new information quickly but also process it at a deeper level. Sustaining the knowledge of the PEWS is important as it relates to timely detection of clinical condition worsening, which leads to better results of the treatment provided to children. This finding is also consistent with evidence from quality improvement studies showing that the quality of implementation of early warning systems improves with ongoing education/instruction [12, 22, 30].

In contrast, the little improvement observed in the control group despite their regular clinical work is a signal that the practical approach alone does not teach professionals how to master the early warning skills. This is consistent with research studies showing that without the help of formal education, nurses cannot recognize the early signs of patients’ deterioration properly or take timely action to provide care to the patients. The findings presented in Table 3 and Figure 2 demonstrate that the educational intervention significantly improved nurses’ clinical performance in applying the BC PEWS observational checklist. At baseline, most nurses in both the control and intervention groups demonstrated unsatisfactory levels of practice (84.2% and 93.0%, respectively), with no statistically significant difference observed between the groups (*p* = 0.141). This indicates that both groups had similarly limited competencies in identifying and managing pediatric clinical deterioration prior to the intervention. These results are consistent with those reported by Ebraheim et al. [11], who highlighted that inadequate training, limited clinical exposure, and inconsistencies in system implementation contribute to nurses’ difficulties in effectively applying early warning systems.

As illustrated in Table 6, a positive and statistically significant correlation between nurses’ knowledge of PEWS and their performance on the BC PEWS observational checklist at follow‐up in the intervention group (*r* = 0.317, *p* = 0.016). This implies that an increase in theoretical knowledge, led to an improvement in clinical performance particularly in terms of detection and intervention in relation to early signs of deterioration. The control group showed a weak correlation (*r* = 0.207, *p* = 0.123), which means that knowledge cannot be implemented in clinical practice if the individuals have not undergone any form of training. This is consistent with previous literature that highlighted the role of training in nurses’ ability to identify early signs of deterioration and utilize PEWSs. For example, according to Chapman et al. [22], training in PEWS increased the success of the detection of physiological changes among nurses. Overall, the findings indicate that it is important to combine theoretical training with practical training when reinforcing the effectiveness of early warning systems. Moreover, the observed association between knowledge and performance in intervention groups suggested that all advanced information regarding clinical deterioration instruments must be gained by them, not only about scoring systems but also in terms of the ability to understand various clinical symptoms in connection with the patient’s problems. Parshuram et al. [30] mentioned the high value of nurses’ clinical judgment and situational awareness in their study of Bedside PEWS. Educational interventions provide training in these skills, which can be developed by means of training in pattern recognition, reinforcing the rules of escalation, and increasing motivation in decision‐making.

Furthermore, the positive progress noticed in the interventional group coincides with findings from nations with lower or middle‐income levels, showing that taking part in PEWS training leads to better results in terms of safety for children. As an illustration, Agulnik et al. [31] observed that implementing PEWS, following staff training in Guatemala, made it possible to detect serious cases faster and decrease the incidence of accidental transfers to the ICU. Likewise, Ebraheim et al. [11] implies that education based on early warning score (EWS) resulted in a considerable increase in nurses’ competency in cases of pediatric deterioration.

The moderate strength of correlation between the intervention group (*r* = 0.317) underscores the multiplicity of factors contributing to clinical performance. Although knowledge is vital, the successful application of PEWS requires the impact of several factors such as workload, nurse‐to‐patient ratios, experience level, as well as the organizational context of the healthcare institution. Roland et al. (2020) mentioned that the effectiveness of an early warning system is heightened when it operates in an atmosphere of safety culture with proper escalation protocols and effective communication with other professionals involved. Thus, the transformative educational intervention used in this research was likely responsible for obtaining knowledge as well as increasing nurses’ confidence in action based on early warning indicators.

According to the results of the regression analysis (Tables 7 and 8), important predictors of nurses’ knowledge at follow‐up were found to be educational level, work experience, and BC PEWS checklist. These findings point to the complexity of the process of becoming knowledgeable after the educational intervention and indicate that both individual and practice‐related factors have an influence on the outcomes of the educational process. The positive correlation between education and knowledge level (*β* = 0.259, *p* = 0.032) indicates that nurses who received good education are better at grasping the theoretical aspects of the PEWS. It is a well‐known fact that getting higher education helps strengthen critical thinking skills, clinical reasoning, and applying evidence‐based practice.

Previous studies have demonstrated that receiving advanced education correlates with a higher level of competence among nurses [33, 34]. Similarly, experience emerged as a significant predictor of knowledge (*β* = 0.217, *p* < 0.001), indicating the value of learning through experience, in practice. The presence of clinical experience will allow nurses to improve their skills in pattern recognition, situation awareness, and decision‐making that are important in detecting warning signs of patient deterioration. Evidence from studies shows that knowledge learned through experience is an indicator of formal education, as more experienced nurses demonstrate higher skills in clinical instruments and complex case management [33]. The finding suggests that knowledge acquisition in nursing is cumulative, which implies learning through clinical practice.

Even though many years of clinical experience have greatly contributed to the acquisition of knowledge, significant positive changes observed after the intervention indicate that experience on its own is not enough to achieve a complete understanding of the PEWS. Systematic PEWS teaching serves as a theoretical basis and practical skills necessary for the proper understanding of clinical deterioration in pediatrics and proper decision‐making, regardless of the number of years nurses have been working [8, 9, 11, 12].

An association between BC PEWS checklist implementation and knowledge (*β* = 0.022, *p* = 0.027) further illustrates the link between cognitive understanding and clinical application. Those nurses who utilized the observational checklist well were more knowledgeable compared to other nurses, which indicates that taking part in clinical activities enhances knowledge learning. The results are consistent with earlier studies that highlighted that the success of the implementation of early warning systems depends on using knowledge and skills because effective assessment and response require both theoretical knowledge and practical skills [35]. The findings support previous studies that showed that educational interventions that incorporate practical practice and simulations are likely to improve the retention of knowledge and practical performance [29].

In contrast, age, gender, and current occupation had no significant influence on knowledge in the present study. Accordingly, this shows that demographic features may have limited impact on learning outcomes compared to areas of expertise. Similar statements have been made in studies of early warning system implementation that have shown that training and clinical exposure, along with support from the workplace, affect the outcomes more than demographic features do. For instance, qualitative data show that education, collaboration, and support by the organization influence nurses’ engagement in early warning system operations [36]. It is essential that these findings indicate various other contextual influences that affect the process of acquiring knowledge. The efficiency of early warning systems is not only determined by individual competencies but is enhanced by the organizational culture, training possibilities, and clear rules of escalation (Roland et al., 2020). Moreover, Flenady et al. [37] pointed out that the extent of adherence to early warning systems is determined by both individual and team‐related factors, including communication, leadership, and institutional support.

In general, the results of the regression analysis can help understand the need for implementing different interventions. Thus, while training programs are important, they might be more effective when complemented by reinforcing clinical experiences and organizational support. The fact that education, experience, and clinical performance were identified as significant predictors highlights the importance of providing competency‐based nursing education and training. This way, nurses’ clinical performance can be improved through PEWS training based on their professional background [15]. The approach is critical to ensure the best outcomes in terms of knowledge retention and effective use of the early warning system to improve safety among pediatric patients. This outcome is in line with the recommendations on the role of pediatric nurses worldwide, which regard training in the use of this system as an essential competency for all family members [31].

## 5. Implications for Nursing Management and Recommendations

The results of this study have major importance for labor force nursing management and the development of the workforce within the nursing profession. It is recommended that nursing managers integrate the education related to the PEWS into their orientation programs, systems of professional development, and competence assessments so that pediatric nurses work with the necessary knowledge and skills for the early detection of clinical deterioration. It is suggested that medical organizations implement universal PEWS documentation processes and appropriate tools, which will evaluate patients, communication, and problem‐solving uniformly within pediatric hospitals.

Moreover, it is advised that hospital administration supports competence‐based training programs incorporating teaching by means of simulation, teaching based on cases, and training for nurses conducted periodically. It is also important that organizational management creates some supportive systems within which PEWS may be implemented successfully and effectively, along with the proper protocols to be followed in case of issues arising related to PEWS implementation. Such activities can contribute to improved personnel quality, patient safety, and children’s medical care.

From the point of view of workforce management, the importance of educational level and work experience for acquiring knowledge and improving performance should motivate nursing managers to look for effective development programs meeting the needs of nurses. The assessment of PEWS competencies should occur as part of quality improvement and clinical management processes.

Further studies should focus on the long‐term influences of PEWS training on the work results of medical personnel, as well as patient safety and results of quality management implementation.

## 6. Conclusion

This study proved that conducting a teaching session on the PEWS can improve students’ knowledge and skills about early recognition and management of pediatric patients’ deterioration. The study results show that education programs on the topic can lead to sustained CQI (continuous quality improvement). Therefore, pediatric nurses’ theoretical knowledge and practical skills need to be supported by structured PEWS training to enable them to perform better after undergoing the intervention. Individual factors such as academic qualifications and years of experience may determine one’s learning needs, but every nurse requires formal PEWS training before engaging in practice. Including the course in orientation, employment, and professional development programs can help achieve positive patient outcomes by enhancing the early detection of deterioration among children and appropriate interventions to improve care.

### 6.1. Limitations

In addition to their positive aspects, the study has several limitations that need to be considered. First, the quasi‐experimental nature of the research and the convenience sampling strategy may affect the study’s external validity, limiting its applicability to a wider population of nurses and clinical settings. Second, the study’s reliance on observational checklists to evaluate nurses’ performance raises questions about inter‐rater reliability and potential biases in assessments. Finally, the relatively brief follow‐up period did not allow the researchers to assess the long‐term impact of the intervention on participants’ knowledge and performance. Future studies on PEWS should utilize random sampling strategies, include a larger and more diverse sample, employ a mixed‐methods design, and extend the follow‐up period to evaluate the intervention’s sustainability and fidelity.

**Nomenclature**
PEWS: Pediatric Early Warning System
PICU: Pediatric intensive care unit
MEWS: Modified Early Warning Score
BC PEWS: British Columbia Pediatric Early Warning System

## Author Contributions

Zeinab Elsayed Hafez Elsayed, Rabia H. Haddad: conceptualization and investigation; Amal A. Elbilgahy, Hend Reda Ali Elkest: methodology and data curation; Rabia H. Haddad, Ashraf J. Abuejheisheh: data curation and formal analysis; Mai Hassan Hassan El‐Sharkawy: methodology and writing–review and editing; Zeinab Elsayed Hafez Elsayed: resources and formal analysis; Sohaib Abdelhaq Aboskeen: writing–original draft; Ashraf J. Abuejheisheh, Mai Hassan Hassan El‐Sharkawy: supervision and data curation; Rabia H. Haddad, Ashraf J. Abuejheisheh: supervision and investigation.

## Funding

The authors report that no financial support, grants, or external funding were received for the preparation or publication of this manuscript.

## Ethics Statement

Ethical authorization for the research was granted by the Scientific Research Ethics Committee at the Faculty of Nursing, Tanta University (Approval No.: 736‐7‐2025). The study protocols presented no physical harm or discomfort to the subjects. Participant privacy and the confidentiality of all gathered data were rigorously preserved. Before initiating data collection of data, written informed consent was secured from every enrolled nurse, who was explicitly notified of their right to discontinue if they faced any consequences. All procedures involving human participants fully adhered to the ethical standards set by the institutional and national committees governing human experimentation, alongside the principles outlined in the Declaration of Helsinki (1964) as revised in 2009 and its subsequent updates.

## Consent

The authors have nothing to report.

## Conflicts of Interest

The authors declare no conflicts of interest.

## Data Availability

The data underlying the findings of this study are not publicly archived but can be made available by the corresponding author upon reasonable request.
